# Cell Surface GRP94 as a Novel Emerging Therapeutic Target for Monoclonal Antibody Cancer Therapy

**DOI:** 10.3390/cells10030670

**Published:** 2021-03-17

**Authors:** Ji Woong Kim, Yea Bin Cho, Sukmook Lee

**Affiliations:** Biopharmaceutical Chemistry Major, School of Applied Chemistry, Kookmin University, Seoul 02707, Korea; jwk7853@kookmin.ac.kr (J.W.K.); yeriel1201@kookmin.ac.kr (Y.B.C.)

**Keywords:** GRP94, cancer, therapeutic target, therapy, monoclonal antibody

## Abstract

Glucose-regulated protein 94 (GRP94) is an endoplasmic reticulum (ER)-resident member of the heat shock protein 90 (HSP90) family. In physiological conditions, it plays a vital role in regulating biological functions, including chaperoning cellular proteins in the ER lumen, maintaining calcium homeostasis, and modulating immune system function. Recently, several reports have shown the functional role and clinical relevance of GRP94 overexpression in the progression and metastasis of several cancers. Therefore, the current review highlights GRP94’s physiological and pathophysiological roles in normal and cancer cells. Additionally, the unmet medical needs of small chemical inhibitors and the current development status of monoclonal antibodies specifically targeting GRP94 will be discussed to emphasize the importance of cell surface GRP94 as an emerging therapeutic target in monoclonal antibody therapy for cancer.

## 1. Introduction

Cancer, the second leading cause of death behind cardiovascular disease, remains a major global public health problem [1]. Overall, the burden of cancer incidence and mortality has continued to increase rapidly worldwide. According to the International Agency for Research on Cancer, an estimated 19.3 million new cancer cases and 10 million cancer deaths occurred in 2020 [2]. The recent increase in cancer incidence has been associated with various factors, such as cigarette smoking, urbanization and the associated pollution, dietary changes, and the psychological stress of modern society [3,4,5]. Over the past several decades, remarkable developments in science and technology, including high-throughput screening, structure-based optimization for drug design, and state-of-the-art technology based on modern biochemistry, cell biology, and molecular biology, have improved the discovery of drugs against cancers [6,7,8,9,10,11]. As of November 2020, approximately 7000 human drug products have been approved by the United States Food and Drug Administration (USFDA) [12]. However, numerous medical unmet needs for successful cancer therapy still exist.

The traditional and most widely used approaches for treating cancer include chemotherapy, radiation therapy, and surgery [13]. Conventional chemotherapy, involving a single drug (single-agent chemotherapy) or several (combination therapy) drugs, usually targets fast-growing cancer cells [14]. Unfortunately, chemotherapy also concomitantly targets normal cells in, for example, hair follicles, bone marrow, and digestive tract, thereby resulting in severe adverse effects, including hair loss, fatigue, nausea, diarrhea, vomiting, anemia, and bone marrow suppression [15,16,17,18]. To overcome the mentioned drawbacks of traditional cancer therapies, monoclonal antibody-based targeted therapy for selective elimination of cancer cells has emerged.

Monoclonal antibody therapy is a form of immunotherapy that uses monoclonal antibodies (mAbs) to bind to a specific target antigen. Since the remarkable development of recombinant DNA technology, more than 570 therapeutic mAbs have been globally studied in clinical trials, and 79 therapeutic mAbs have received USFDA approval. In particular, around 30 USFDA-approved mAbs have been on the market to treat a variety of hematological and solid cancers [19]. Among these, rituximab (Mabthera; Rituxan) was the first mouse/human chimeric mAb approved by USFDA in 1997 to treat relapsed or refractory CD20-positive non-Hodgkin’s lymphoma [20]. However, despite the clinical use of numerous anticancer therapeutic antibodies, a limited number of their therapeutic targets, including CD20, vascular endothelial growth factor-A (VEGF-A), epidermal growth factor receptor (EGFR), human epidermal growth factor receptor 2 (HER2), programmed cell death protein-1 (PD-1), and programmed death-ligand 1 (PD-L1), have currently been identified [21,22,23,24,25]. Some examples include ofatumumab, ocrelizumab, ibritumomab tiuxetanand, and rituximab for CD20-targeted mAbs; bevacizumab, ranibizumab, and brolucizumab for VEGF-A-targeted mAbs; cetuximab, panitumumab, and necitumumab for EGFR-targeted mAbs; trastuzumab, and pertuzumab for HER2-targeted mAbs; nivolumab, pembrolizumab, and cemiplimab for PD-1-targeted mAbs; lastly, atezolizumab, avelumab, and durvalumab for PD-L1-targeted mAbs [21,22,26,27,28,29,30,31,32,33,34,35,36,37,38,39,40,41,42]. As such, identifying novel therapeutic targets and understanding the molecular mechanism of metastatic cancer cells are essential for overcoming unmet medical needs in current cancer therapy.

Therefore, the current review highlights GRP94’s physiological roles in cells and its roles and relevance in cancers to clarify its pathological mechanisms in cancer progression and metastasis. Additionally, outlining the current status of GRP94-targeting inhibitors will provide insight into unmet medical needs for monoclonal antibody therapy.

## 2. The Structure and Physiological Roles of GRP94 in Cells

### 2.1. The Structure of GRP94

GRP94 is an endoplasmic reticulum (ER)-resident member of the heat shock protein 90 (HSP90) family, a paralog of cytosolic HSP90 [43]. It is also known as tumor rejection antigen 1 (TRA1), gp96, heat shock protein 90 kDa beta member 1 (HSP90B1), and endoplasmin [44,45,46]. Many different groups have discovered that GRP94, as a cell protein, is strongly induced by glucose starvation [47]. It is also a major calcium-binding protein in the ER and the most abundant ER-resident protein [48,49]. Several methods for determining the structure of GRP94, including X-ray crystallography, electron microscopy, and small-angle X-ray scattering, have revealed that it forms a “twisted V”-shaped dimeric structure in physiological conditions [50,51]. The molecular structure of GRP94 comprises four main conserved domains, including an N-terminal domain (NTD), a charged linker region (CR), a middle domain (MD), and a C-terminal domain (CTD). 

**Figure 1:**
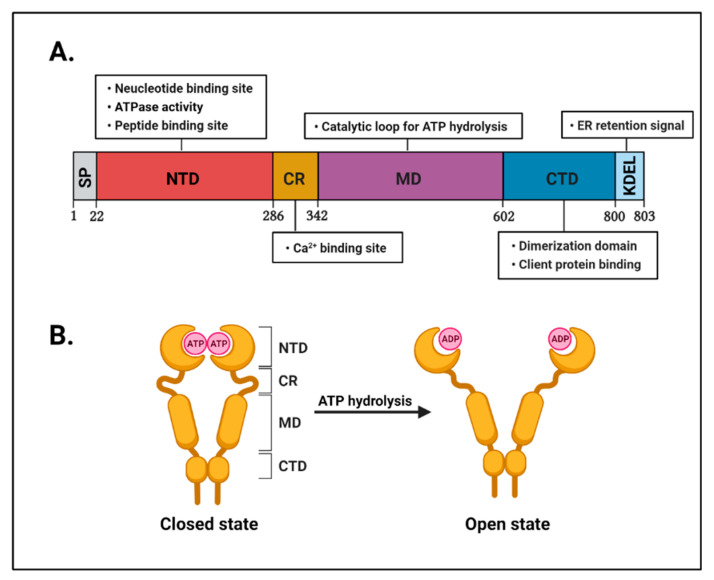
Structural feature of GRP94. (**A**) A schematic representation of the domain organization of human GRP94. The first 21 sequences are signal peptide sequences (gray) displayed as SP. Next, the N-terminal domain (NTD; red) plays an important role in ATPase activity and contains a nucleotide and peptide binding site. The charged linker region (CR) contains a calcium-binding site (orange). The middle domain (MD; purple) contains a catalytic loop for ATP hydrolysis. The C-terminal domain (CTD; blue) plays an important role in dimerization and client protein binding. KDEL (light blue), the C-terminal tetrapeptide of GRP94, serves as the endoplasmic reticulum (ER) retention/retrieval ligand for the KDEL receptor. (**B**) Schematic representation of the GRP94 structure. The conformational change in GRP94 occurs through ATP binding and ATP hydrolysis. GRP94 changes its state from “closed” to “open” through ATP hydrolysis.

The NTD (amino acid residues 22–285) has an adenosine triphosphate (ATP)-binding pocket that is essential for ATPase activity [52]. Thus, the NTD has been used as a major target for GRP94 inhibitors such as geldanamycin (GDA) and radicicol (RDC) [53,54]. These are natural product inhibitors and ATP-competitive N-terminal inhibitors. The CR (amino acid residues 286–341) is a short dynamic region that connects the NTD and the MD. This domain is essential for controlling ATP hydrolysis activity in cooperation with the MD [55]. Furthermore, the CR also contains calcium-binding sites required for intracellular calcium homeostasis [56]. The MD (amino acid residues 342–601) contains the catalytic loop for ATPase activity and interacts with the NTD for ATP hydrolysis [57]. Lastly, the CTD (amino acid residues 602–803) is critical for not only providing a homodimer interface for the dimer formation of GRP94 but also containing a client protein-binding site for GRP94 [58]. In addition, the CTD ends with a KDEL tetrapeptide sequence that is critical for the retention of GRP94 in the ER lumen [59] (Figure 1).

### 2.2. Physiological Role of GRP94 in Cells

GRP94 was first identified as a cell protein strongly upregulated in response to glucose starvation in the ER [60]. Furthermore, in normal cells, GRP94 is also specifically upregulated by a variety of stress conditions that perturb ER functions, including oxidative stress, ER calcium depletion, accumulation of misfolded proteins, and glucose starvation [61,62]. GRP94 shares many biochemical features, such as domain structure and ATPase activity, with other HSP90 family members [63]. Thus far, several studies have suggested that GRP94 plays multifunctional roles in physiological conditions.

#### 2.2.1. GRP94 as a Molecular Chaperone

**Figure 2:**
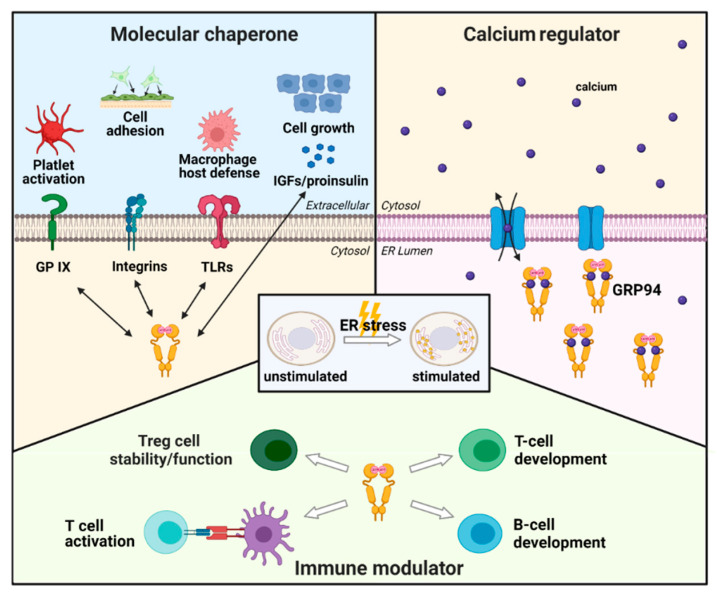
Schematic representation of the physiological roles of GRP94. GRP94 is a molecular chaperone upregulated by ER stress. Under stress conditions, GRP94 accelerates its function as a molecular chaperone. Client proteins of GRP94 include toll-like receptors (TLRs), glycoprotein (GP) IX subunit, insulin-like growth factors (IGFs), proinsulin, and integrins. These interactions are closely associated with direct function in macrophage host defense, activation of platelets in blood clotting, cell growth, and cell adhesion. Calcium regulation is another physiological function of GRP94, one of the major calcium-binding proteins in the ER regulating calcium homeostasis. Lastly, GRP94 is a multifunctional immune modulator. As demonstrated, GRP94 is essential in early T- and B-cell development while also regulating Treg cell stability and immunosuppressive function. GRP94 activates T-cells through peptide binding.

GRP94 mainly serves as an ER-resident molecular chaperone that physically interacts with and directs the folding and assembly of several secreted and membrane client proteins [64,65]. Eventually, it participates in regulating various functions, including cell growth, adhesion, and immunity (Figure 2).

Notably, Melnick et al. identified newly synthesized and unassembled immunoglobulin (Ig) heavy or light chains as the first GRP94 client proteins related to immunity [66]. They suggested that GRP94 assists in the correct folding and assembly of Ig subunits. Meanwhile, Randow et al. reported that GPR94 forms a complex with toll-like receptors (TLRs) [67]. These pattern recognition receptors recognize immune system pathogens and play a vital role in the folding, assembly, and export of TLRs, including TLR1, TLR2, and TLR4. After generating macrophage-specific GRP94 knockout mice and analyzing macrophage responses to TLR agonists, Yang et al. found that GRP94-deficient macrophages failed to respond to ligands for plasma membrane TLRs, suggesting the importance of GRP94 in regulating TLR responses and host defense to bacterial infection [68]. Additionally, Staron et al. revealed that the selective binding of GRP94 to the glycoprotein (GP) IX subunit plays a key role in the assembly of the platelet GP I b-IX complex, critical for platelet activation in blood clotting [69].

Apart from immune client proteins of GRP94, insulin-like growth factors (IGFs) and integrins have also been identified as major GRP94 client proteins. Ostrovsky et al. reported that GRP94 interacts physically and transiently with pro-IGF-II intermediates, while its activity is essential for the secretion of active IGF-II, thereby establishing IGF-II as a client of GRP94 [70]. By using muscle tissue-specific conditional knockout of GRP94, Barton et al. showed that GRP94 plays an important role in muscle and whole-body growth by controlling IGF-I secretion and production [71]. In addition, Ghiasi et al. reported that GRP94 participates in proinsulin production as a molecular chaperone. They demonstrated that GRP94 forms a complex with proinsulin in INS-1E rat pancreatic β-cells. Moreover, the study also revealed that GRP94 lentiviral short hairpin RNA (shRNA)-induced knockdown or CRISPR/Cas9-induced knockout significantly reduced proinsulin production in INS-1E cells [72]. Furthermore, Wu et al. reported that amino acid residues 652–678 of GRP94 and, more specifically, Met^658^ and Met^662^ are critical for binding and chaperoning integrin αL [73]. Randow et al. showed that the inducible deletion of GRP94 reveals its critical role in the expression of several integrins, including αL, α4, and αM [67]. Furthermore, Chen et al. reported that liver-specific GRP94 knockout in mice resulted in disruption of cell adhesion in liver progenitor cells due to integrin β1 loss. [74]. These studies demonstrate that the interaction between GRP94 and integrins is closely related to cell adhesion.

Lastly, thyroglobulin, bile-salt-dependent lipase, glycoprotein A repetitions predominant, and low-density lipoprotein receptor-related protein 6 were also identified as client proteins of GRP94 [75,76,77,78].

#### 2.2.2. GRP94 as a Calcium Regulator

The calcium (Ca^2+^)-binding proteins in the ER not only provide a large Ca^2+^ storage capacity that enables the ER to accumulate high levels of free and bound Ca^2+^ but also regulate the concentration of intracellular Ca^2+^ for the activation of several signaling pathways and physiological responses in response to a variety of cellular stimuli [79,80] (Figure 2). Several lines of evidence suggest that GRP94 is one of the major Ca^2+^-binding proteins in the ER lumen and that it plays a key role in regulating cellular Ca^2+^ homeostasis [48]. Drummond et al. reported that the Ca^2+^ ionophore A23187 depletes intracellular Ca^2+^ stores and upregulates GRP94 mRNA expression in hamster fibroblasts [81]. Each GRP94 molecule has 15 putative Ca^2+^-binding sites, among which 4 and 11 have higher (K_D_ ~2 μM) and lower (K_D_ ~600 μM) affinity sites, respectively [48]. Other groups have also shown that purified GRP94 can bind 280 nmol of Ca^2+^ per mg protein [82]. Furthermore, Biswas et al. showed that the N-terminal portion of GRP94 contains at least one high-affinity Ca^2+^ binding site within its charged linker domain. The same paper also demonstrated that GRP94 knockout embryonic stem cells stopped growing in Ca^2+^-depleted medium, suggesting the unique role of GRP94 in Ca^2+^ homeostasis [56]. Additionally, Vitadello et al. reported that GRP94 overexpression promoted a significantly slower increase in intracellular Ca^2+^ in Ca^2+^ ionophore A23187-exposed muscle cell lines than in control cells, suggesting GRP94′s role as an intracellular Ca^2+^ regulator [83]. Similarly, Bando et al. also reported that exposure to Ca^2+^ ionophore A23187 increased GRP94′s expression in human neuroblastoma SH-SY5Y cells and that GRP94 could suppress A23187-induced cell death in SH-SY5Y cells, implying its Ca^2+^ buffering function [84].

#### 2.2.3. GRP94 as an Immune Modulator

Apart from its chaperone function, GRP94 seems to play an important role in modulating the immune system (Figure 2), with several lines of evidence supporting this notion. Accordingly, Staron et al. reported that GRP94 knockout mice blocked T- and B-cell lymphopoiesis, indicating its central role in B- and T-cell development [85]. Zheng et al. demonstrated that GRP94 is important for stimulating dendritic cell maturation and inducing efficient T-cell priming [86]. Furthermore, Suto et al. showed direct evidence that assembled complexes of GRP94 and synthetic peptides can be presented again by an antigen-presenting cell (APC) to promote T-cell activation [87]. Biswas et al. also identified that the N-terminal fragment (amino acids 1–355) of GRP94 is responsible for peptide presentation to T-cells [56]. Thus, the studies above provide definitive evidence that GRP94 can direct peptides to MHC class I in antigen presentation and induce APC maturation and activation [88]. Tramentozzi et al. showed that purified GRP94 stimulates Ig secretion and PBMC proliferation, indicating that GRP94 can activate the humoral response via a cytokine-like, cell-mediated mechanism [89]. Lastly, Zhang et al. demonstrated that GRP94 disruption in regulatory T-cell (Treg)-specific GRP94 knockout mice impaired the suppressive function of Treg cells in vivo and promoted the development of fatal inflammatory diseases. The same study also reported that the Treg cell lineage exhibited instability and underwent conversion into IFN-γ-producing pathogenic T-cells in the absence of GRP94, suggesting that GRP94 may play a central role in Treg cell stability and immunosuppressive functions [90].

## 3. Role and Relevance of GRP94 in Cancer

### 3.1. Clinical Relevance of GRP94 in Cancer

Many studies have demonstrated that under stress conditions, GRP94 assists in the folding of newly synthesized polypeptides and prevents the aggregation of unfolded or misfolded proteins in the ER lumen [91]. Tumors particularly exhibit a wide range of stress conditions, including hypoxia, redox homeostasis changes, altered cell metabolism, acidosis, and increased cell proliferation and protein synthesis, all of which can trigger ER stress [92,93,94].

Reports have shown that GRP94 mRNA is upregulated in several types of cancer tissues, including liver cancer, breast cancer, esophageal cancer, and glioma tissues [95,96,97,98]. Furthermore, several immunohistochemical studies have revealed that GRP94 protein is highly overexpressed in various cancers, including breast, lung, colorectal, oral, esophageal, and gastric, suggesting a strong relationship with cancers [97,99,100,101,102,103]. Several among the mentioned cancers have shown an inverse correlation between GRP94 overexpression and patient survival. For instance, Liu et al. reported that patients with breast cancer tissues expressing high GRP94 had a statistically significantly shorter survival time than those with a low GRP94 expression [96]. Moreover, multiple studies have suggested that GRP94 may be a potential poor prognostic factor in various types of cancers, including lung, gastric, colorectal, and esophageal cancers [100,104,105,106]. In summary, available evidence suggests that GRP94 is closely associated with cancer progression and metastasis.

### 3.2. Role of GRP94 in Cancer Progression and Metastasis

During the multistep development of human tumors, cancer hallmarks include uncontrolled cell proliferation, tumor angiogenesis, invasion, and metastasis [107,108]. Accumulating evidence has revealed that GRP94 is strongly associated with increased cancer proliferation. Several in vitro experiments have demonstrated that GRP94 knockdown in cancer cells promoted growth reduction. For instance, Duan et al. reported that GRP94 knockdown in lung cancer cells inhibits its proliferation and promotes cell apoptosis by increasing caspase-7 and C/EBP homologous protein levels [100]. Moreover, Huang et al. reported that GRP94 knockdown in two different esophageal cancer cell lines using short hairpin RNA (shRNA) promoted more than 50% growth inhibition [106]. Similarly, multiple in vitro studies demonstrated that GRP94 knockdown facilitated growth inhibition in various cancer cell lines, such as gastric cancer, breast cancer, and colorectal cancer cells [99,109,110]. Another study using an in vivo xenograft mouse model showed that subcutaneous injection of GRP94-deficient hepatocellular carcinoma (HCC) cells resulted in significant tumor growth reduction [109].

Tumor angiogenesis is a vital process wherein new blood vessels are formed to properly establish a supportive microenvironment rich in oxygen and nutrients, necessary for optimal growth. Zhang et al. reported considerable growth suppression after orthotopically injecting a GRP94-knockdown melanoma cell line into mice. Further mechanistic studies demonstrated that GRP94 depletion reduced VEGF-A expression, inhibiting tumor-associated angiogenesis [110].

Subsequently, an increasing number of reports have shown a strong relationship between GRP94 and cancer invasion and metastasis. Accordingly, Calderon et al. observed a significant decrease in invasion following GRP94 knockdown in MDA-MB-231, a highly aggressive human breast cancer cell line [111]. Wei et al. also reported that GRP94 knockdown in GRP94 shRNA-treated HCCs inhibited their invasive characteristics, including wound healing, migration, and invasion. Further analysis revealed the inhibition of the chaperonin-containing TCP1 subunit 8/c-Jun/epithelial–mesenchymal transition (CCT8/c-Jun/EMT) cascade in GRP94 shRNA-treated HCCs attenuated its invasive characteristic [112]. Moreover, the influence of GRP94 on cancer cell invasion may be explained by the fact that its client proteins include cell adhesion components, such as integrins. Recently, Hong et al. demonstrated a cell-permeable peptide that competitively inhibited the interaction between GRP94 and integrins, blocking cell invasion in leukemia [113]. Furthermore, Wang et al. reported that GRP94 expression was significantly higher in poorly differentiated colon cancers with metastasis than in well-differentiated cancers without metastasis [114]. Additionally, a statistical analysis study by Pamplona et al. demonstrated significant associations between brain metastasis progression and high GRP94 expression [115].

### 3.3. Role of GRP94 in Tumor Resistance

Tumor resistance to conventional therapies has remained a major challenge for successful cancer treatment [116]. Thus, discovering factors predicting cancer resistance is crucial for screening and improving adjuvant therapies for patients with cancer and preventing unnecessary treatment side effects. Multiple studies have suggested that GRP94 participates in tumor radio- and chemoresistance. Accordingly, Lin et al. observed that GRP94 was overexpressed in radioresistant head and neck cancer cells, and using siRNA against GRP94 restored radiosensitivity in the same cancer cell lines [117]. Moreover, Kubota et al. found that cervical cancer cells with increased GRP94 expression were more resistant to X-ray, while Wang et al. reported that incubation of malignant cells with chemotherapeutic agents, such as 5-fluorouracil, cisplatin, and paclitaxel, upregulated GRP94 expression [118,119]. Additionally, Zhang et al. reported that GRP94 was associated with decreased sensitivity to doxorubicin in ovarian carcinoma cell lines. Similarly, Calderon et al. reported that siRNA-induced knockdown of GRP94 expression in human breast cancer cells helped increase their sensitivity to doxorubicin [111].

## 4. The Development of GRP94-Specific Inhibitors

### 4.1. GRP94 Small Molecule Inhibitors for Cancer Therapy

Thus far, significant attention has been paid to GRP94 as a potential therapeutic target for GRP94 small molecule inhibitors [120]. It is particularly well-known that GRP94-induced protein folding depends on conformational changes in the chaperone driven by cycles of ATP binding and hydrolysis [121]. Thus, several GRP94 small molecule inhibitors have been developed to inhibit ATP binding and hydrolysis competitively [122,123]. At present, GRP94 inhibitors can be classified into three classes, namely, benzoquinone ansamycin, resorcinol, and purine [124].

#### 4.1.1. Benzoquinone Ansamycin Class

GDA, a benzoquinone ansamycin, was originally isolated as a natural product inhibitor of both HSP90 and GRP94, with potent and broad anticancer properties [125]. Reports have shown that GDA competitively binds to the ATP-binding pocket in the NTD of GRP94 and inhibits its chaperone activity via downregulation of ATPase activity, resulting in the effective and potent killing of cancer cells [126]. However, owing to the substantial hepatotoxicity and unsatisfactory solubility of GDA, it remains a poor clinical candidate [127,128]. Nonetheless, subsequent modifications have been made to optimize GDA and improve its therapeutic index.

GDA derivative 17-allylamino-17-demethoxygeldanamycin (17-AAG) was the first HSP90 inhibitor to enter clinical trials [129,130]. 17-AAG received considerable attention given its lower toxicity than GDA and potent therapeutic efficacy for both hematologic and solid tumor animal models over multiple clinical trials. Like GDA, 17-AAG binds to HSP90 and GRP94 with similar affinities and competitively inhibits ATP binding [131]. However, this product was discontinued due to poor pharmaceutical properties, namely, water insolubility, such that it required the addition of organic additives, such as dimethyl sulfoxide and polyoxyl castor oil (cremophor), to achieve aqueous solution solubility [132]. When administered to patients, these organic solvents may cause several adverse events, including nausea, vomiting, hypersensitivity reactions, and anaphylaxis. Furthermore, clinical trials using 17-AAG together with these additives may have masked the true maximum tolerable dose of 17-AAG in patients and misled physicians when scheduling the optimal dosing required to help manage toxicities [133,134]. Therefore, more extensive effort has been directed toward developing novel, water-soluble HSP90 inhibitors.

Notably, 17-desmethoxy-17-*N*,*N*-dimethylaminoethyl amino geldanamycin (Alvespimycin; 17-DMAG) is a water-soluble GDA derivative with reduced hepatotoxicity. While 17-DMAG differs from 17-AAG in the position 17 side chain of the ansa ring, it exhibits similar therapeutic efficacy and mode of action. Surprisingly, 17-DMAG is more soluble in aqueous media, such as saline, compared to 17-AAG, resulting in greater bioavailability [135]. Unfortunately, this compound did not show sufficiently promising activity in phase II clinical testing, for which further development has been abandoned [136].

#### 4.1.2. Resorcinol Class

RDC is a natural macrocyclic antifungal antibiotic originally isolated from *Monosporium bonorden* in 1953 [54,137]. RDC competitively binds to the ATP-binding site of HSP90 and GRP94 and has been found to induce apoptosis even in 17-AAG-resistant cancer cells [138]. While RDC is the most potent HSP90 inhibitor in vitro, it has failed to be effective in animal models due to its unstable epoxy group [139]. Moreover, radamide (RDA), a chimera of RDC and GDA, had been initially designed to favorably interact with a unique hydrophobic-binding pocket, exclusive to GRP94, but it did not show higher selectivity for GRP94 (Kd = 0.52 μM) over HSP90 (Kd = 0.87 μM) [140].

NVP-AUY922 is a resorcinol-derived synthetic molecule discovered using a structure-based drug designing strategy. NVP-AUY922 had an IC_50_ value of 535 ± 51 nM against GRP94, indicating weaker potency than HSP90 [141,142]. This molecule, developed by Novartis, reached phase II clinical trials to treat patients with refractory gastrointestinal stromal or pancreatic cancers. However, studies were discontinued after it failed to show clinically significant effectiveness at the maximum tolerable dose [143]. Several lines of evidence have led us to speculate that the insufficient response of HSP90 inhibitors in clinical trials may result from chemoresistance caused by the increased expression of HSP70. For example, multiple studies have demonstrated that HSP90 inhibitors such as 17-DMAG and NVP-AUY922 upregulate the expression of HSP70 in vitro or in vivo [144,145,146,147]. Ghoshal et al. reported that siRNA-mediated HSP70 knockdown sensitizes the apoptosis of HEL human acute myeloid leukemia cells to 17-DMAG [148]. Furthermore, Kühnel et al. also reported that siRNA-mediated downregulation of HSP70 significantly increased the potency of NVP-AUY922 to H1339 lung cancer cells. [149]. However, despite these current studies, there remains a need for more detailed studies to further investigate the molecular mechanism of HSP90 inhibitors.

#### 4.1.3. Purine Class

PU-H71, first discovered by Memorial Sloan-Kettering Cancer Centre, has undergone a phase I clinical trial by Samus Therapeutics. However, toxicity-related issues (life-threatening grade IV hematologic toxicities) halted further clinical evaluations [150,151].

5′-*N*-ethylcarboxamidoadenosine (NECA) was originally identified as a GRP94-selective inhibitor. However, a recent report by Liu et al. revealed that NECA inhibits multiple HSP90 proteins, including GRP94, HSP90α, HSP82, and TRAP1 [152]. Although NECA interacts preferentially with GRP94, using the NECA scaffold for further inhibitor development has been limited because NECA is also a potent agonist of several cellular adenosine receptors [153].

BIIB021, initially developed by Conforma Therapeutics (currently Biogen Idec) through a structure-based design based on the purine scaffold, is currently undergoing a phase II clinical trial [154]. Accordingly, Ernst et al. reported that BIIB021 inhibited not only GRP94 (Kd = 143 nM) but also HSP90 (Kd = 2 nM). Thus far, known adverse effects of BIIB021 include syncope, dizziness, fatigue, hyponatremia, and hypoglycemia [155]. Nevertheless, since this agent seems to elicit therapeutically significant anticancer activity at the clinical level, clinical evaluations are underway.

### 4.2. GRP94 Monoclonal Antibodies for Cancer Therapy

#### 4.2.1. Cell Surface GRP94 in Cancers

GRP94, as a molecular chaperone, promotes proper folding of unfolded or misfolded proteins and suppresses their aggregation in the ER [156]. Despite its role in the ER, multiple studies have also observed GRP94 on the surface of cancers. Accordingly, Li et al. were the first researchers to exhibit cell surface GRP94 expression through immunofluorescence staining from nonpermeabilized SK-BR-3 human breast cancer cells [157]. Over the following years, reports have shown that cell surface GRP94 is highly expressed in various human cancer cell lines, such as SLR21 renal cancer, PANC10.05 pancreatic cancer, OVCAR3 ovarian cancer, DU-145 prostate cancer, WM1158 melanoma, and HCT-116 colorectal cancer cells [110,158,159]. Moreover, Melendez et al. demonstrated that cell surface GRP94 is especially expressed in MCF-7 and AU565 malignant breast cancer cells and not in MCF-10A and HMEC nonmalignant breast cancer cells [160].

**Figure 3:**
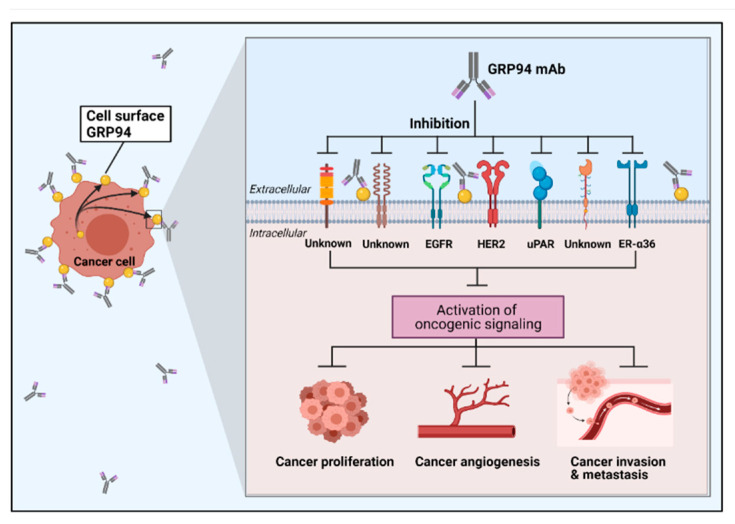
Schematic representation of cancer cell surface GRP94-targeting antibody, with its pathological role in the cancer microenvironment. GRP94 is highly expressed on the cancer cell surface. Several receptors are known for interacting with cell surface GRP94, and they facilitate cancer development by activating oncogenic signaling. So far, EGFR, HER2, uPAR, and ER-α36 are known transmembrane receptors that require GRP94 for cancer development and activation. However, still undiscovered receptors that interact with cell surface GRP94 to promote cancer development may exist. Targeting cell surface GRP94 in cancer inhibits its interactions, resulting in the reduction of oncogenic signaling transduction, which leads to the inhibition of cancer development.

Studies have shown that cell surface GRP94 in cancer is closely associated with the promotion of cancer cell proliferation, invasion, and metastasis. Accordingly, Li et al. reported that GRP94 specifically interacts with HER2 at the plasma membrane of SK-BR-3 human breast cancer cells. The same paper also showed that overexpression of cell membrane GRP94 promotes cell proliferation and tumor growth by enhancing HER2 dimerization and the downstream signaling pathway. Furthermore, immunohistochemical analysis revealed that HER2 activation correlates with plasma membrane GRP94 expression in patients with HER2-positive primary breast cancer [157]. Hou et al. also reported that elevated cell surface GRP94 was associated with tumor metastasis and recurrence in patients with primary liver tumors. Furthermore, the same paper revealed that GRP94 interacts with urokinase-type plasminogen activator receptor (uPAR) in SK-HEP-1 human hepatoma cells, enhancing cancer cell stability, proliferation, survival, and invasion [161]. Additionally, Yan et al. also demonstrated that GRP94 present at the plasma membrane has a higher N-glycan content than ER-resident GRP94. The cell surface GRP94 forms a complex with HER2 and EGFR in breast cancer cells [158] (Figure 3).

#### 4.2.2. Mouse Monoclonal Antibody

Monoclonal antibodies can be a powerful tool for validating a target protein’s potential therapeutic role for antibody therapy. Regarding GRP94-specific mouse monoclonal antibodies, the following evidence highlights the key role of GRP94 as a potential therapeutic target in cancers. Accordingly, Li et al. reported that cell surface GRP94 interacts with HER2, facilitates HER2 dimerization, and promotes cell proliferation. The same authors further demonstrated that cell surface GRP94-specific mouse antibody blocks GRP94-dependent HER2 dimerization and phosphorylation in SK-BR-3 breast cancer cells. It also suppresses HER2-driven breast cancer cell growth and induces apoptosis in a xenograft animal model [157]. Moreover, a study by Hou et al. reported that GRP94-targeting mouse mAb blocks the interaction between GRP94 and estrogen receptor-α36 (ER-α36) in the plasma membrane of MDA-MB-231 breast cancer cells, which play an important role in breast cancer growth and development. The same study showed that antibody treatment significantly inhibits breast cancer cell growth and invasion in vitro as well as tumor growth in a mouse xenograft model [159]. Another study also found that GRP94 mouse mAb specifically blocks the GRP94–uPAR interaction and inhibits uPAR-driven liver cancer cell growth, survival, and invasion, both in vitro and in vivo [161]. Collectively, these studies suggest that cell surface GRP94 could be a potential therapeutic antibody therapy target for cancers (Figure 3).

#### 4.2.3. Human Monoclonal Antibody

Mouse monoclonal antibodies are often confronted with immunogenicity concerns caused by mouse-derived protein sequences [162,163]. Recent advancements in recombinant DNA technology have facilitated the remarkable development of therapeutic antibodies, including humanized and fully-human antibodies, which may open new avenues for improving patient survival and quality of life [19,164,165]. Sabbatino et al. were the first researchers to develop a human monoclonal antibody-targeting GRP94 named W9. More interestingly, they found that the antibody specifically recognizes the extracellular epitope of GRP94 on the membrane surface of malignant cells but not on normal cells. Furthermore, the W9 antibody could increase and restore sensitivity to v-raf murine sarcoma viral oncogene homolog B1 (BRAF) inhibitors in BRAFV600E melanoma cells that have acquired BRAF inhibitor resistance due to PDGFRα upregulation [166]. The same group also reported that treatment with the W9 antibody significantly suppressed MV3 human melanoma cell line growth in vitro. This effect also reflects the induction of apoptosis and the inhibition of several signaling pathways, including extracellular signal-regulated kinase, protein kinase B, and focal adhesion kinase pathways. Furthermore, they demonstrated that the W9 antibody induced the regression of experimental lung metastasis established in immunodeficient mice via intravenous injection of M21 melanoma cells [167].

Cetuximab is a recombinant mouse/human chimeric monoclonal antibody that targets EGFR to treat patients with EGFR- and wild-type Kirsten rat sarcoma 2 viral oncogene homolog (KRAS)-expressing colorectal cancer [168]. However, it is only effective in approximately 10–20% of patients with colorectal cancer, with other patients showing cetuximab resistance due to gene mutations in downstream EGFR effectors, including phosphoinositide-3-kinase catalytic subunit alpha (PI3KCA), phosphatase and tensin homolog (PTEN), KRAS, and BRAF [164,169]. Another interesting study by our group aimed to isolate antibodies tightly bound to the surface of HCT-116 cetuximab-resistant human colorectal cancer cells. To do so, we intensively performed several rounds of cell panning using phage display technology for antibody selection, followed by the isolation of a single-chain fragment variable (scFv) clone that strongly binds to the surface of HCT-116 cells from the combinatorial human antibody library. After converting the selected scFv clone to immunoglobulin G (IgG) and using peptide mass fingerprinting, we finally identified a 100-kDa target human GRP94 antigen. Furthermore, we demonstrated that the selected IgG antibody potently inhibited the growth of various HT-29, LoVo, HCT-8, and HCT-116 cells and significantly suppressed colorectal cancer growth without severe toxicities in an HCT-116 xenograft mouse model [165]. In the study above, we suggested that cell surface GRP94 may be a potential and novel therapeutic target in cetuximab-resistant colorectal cancers. Although further studies are needed to elucidate our findings, this study also provides substantial evidence that antibody-based targeting of cell surface GRP94 can be effective against GRP94-expressing colorectal cancers.

## 5. Conclusions

Cancer consists of a mixture of multiple and heterogeneous clones of tumor cells with different characteristics. Despite the numerous therapeutic agents developed for cancer therapy over several decades, such as small chemical inhibitors and mAbs, severe adverse effects and drug resistance have remained major obstacles toward better clinical outcomes. Thus, identifying a novel potential therapeutic target that is closely associated with the pathogenesis of cancer progression and metastasis is still an important challenge for new drug discovery and development.

Thus far, several reports have identified GRP94 as a prognostic marker that plays a critical role in cancer cell progression and metastasis. Although there has been considerable effort to develop small molecules that inhibit GRP94 in cancers selectively, several studies were discontinued due to severe adverse effects, such as hepatotoxicity caused by off-target effects, in some clinical trials. At the same time, several murine and human monoclonal antibodies have been generated to evaluate the role of cell surface GRP94 in antibody therapy for cancers. Compared to membrane-permeable small molecules that simultaneously inhibit both GRP94 in the ER lumen and cell surface GRP94, antibody targeting of cell surface GRP94 is speculated to be much safer, considering that high molecular weight antibodies (150 kDa) do not penetrate the plasma membrane. Thus, based on currently available evidence, we suggest that cell surface GRP94 may be a novel potential therapeutic target in antibody therapy for cancers. Furthermore, antibody-based targeting of cell surface GRP94 may be an effective strategy for suppressing tumor progression and metastasis.

## Data Availability

Not applicable.

## Abbreviations

17-AAG	17-Allylamino-17-demethoxygeldanamycin
17-DMAG	17-Desmethoxy-17-*N*,*N*-dimethylaminoethyl amino geldanamycin
AKT	Protein kinase B
APC	Antigen-presenting cell
ATP	Adenosine triphosphate
BRAF	v-raf murine sarcoma viral oncogene homolog B1
BSDL	Bile-salt-dependent lipase
CHOP	C/EBP homologous protein
CR	Charged linker region
CRC	Colorectal cancer
CTD	C-terminal domain
EGFR	Epidermal growth factor receptor
ER	Endoplasmic reticulum
ERK	Extracellular signal-regulated kinase
ER-α36	Estrogen receptor-α36
FAK	Focal adhesion kinase
GARP	Glycoprotein A repetitions predominant
GDA	Geldanamycin
GRP94	Glucose-regulated protein 94
GP	Glycoprotein
HCC	Hepatocellular carcinoma
HER2	Human epidermal growth factor receptor 2
HSP90	Heat shock protein 90
HSP90B1	Heat shock protein 90 kDa beta member 1
IGFs	Insulin-like growth factors
IgG	Immunoglobulin G
LRP6	Lipoprotein receptor-related protein 6
KRAS	Kirsten rat sarcoma 2 viral oncogene homolog
mAbs	Monoclonal antibodies
MD	Middle domain
NECA	5′-N-ethylcarboxamidoadenosine
NTD	N-terminal domain
PD-1	Programmed cell death protein-1
PD-L1	Programmed death-ligand 1
PI3KCA	Phosphoinositide-3-kinase catalytic subunit alpha
PTEN	Phosphatase and tensin homolog
RDA	Radamide
RDC	Radicicol
scFv	Single-chain variable fragment
shRNA	Short hairpin RNA
TLRs	Toll-like receptors
TRA1	Tumor rejection antigen 1
uPAR	Urokinase-type plasminogen activator receptor
USFDA	The United States Food and Drug Administration
VEGF-A	Vascular endothelial growth factor-A
