# Correction: Kim et al. Cell Surface GRP94 as a Novel Emerging Therapeutic Target for Monoclonal Antibody Cancer Therapy. *Cells* 2021, *10*, 670

**DOI:** 10.3390/cells10051198

**Published:** 2021-05-14

**Authors:** Ji Woong Kim, Yea Bin Cho, Sukmook Lee

**Affiliations:** Biopharmaceutical Chemistry Major, School of Applied Chemistry, Kookmin University, Seoul 02707, Korea; jwk7853@kookmin.ac.kr (J.W.K.); yeriel1201@kookmin.ac.kr (Y.B.C.)

In Section 4.1.3 of the published paper, the authors made an incorrect and unsupported statement regarding PU-H71 and a Samus-sponsored Phase 1 clinical trial [1]. The authors wish to acknowledge that:

The cited references do not apply to PU-H71. Moreover, no clinical study with PU-H71 conducted by Samus Therapeutics or others has experienced life-threatening grade IV hematologic toxicities. In fact, PU-H71 is currently be administered in several active Phase 1b clinical studies.

Therefore, the sentences “PU-H71, first discovered by Memorial Sloan-Kettering Cancer Center, has undergone a phase I clinical trial by Samus Therapeutics. However, toxicity-related issues (life-threatening grade IV hematologic toxicities) halted further clinical evaluations [150,151].” were amended to “PU-H71, one of the first discovered epichaperome-complexed HSP90 inhibitors, was developed by the group from Memorial Sloan-Kettering Cancer Center [151]. PU-H71 is highly specific for abnormal cells in that epichaperomes only form in diseased cells and inhibits HSP90 when incorporated into the epichaperome; PU-H71 does not inhibit the HSP90 in normal cells [152]. In addition, several lines of recent studies have shown that, under equilibrium conditions, PU-H71 also inhibits GRP94, a HSP90 paralog [153–155]. Currently, PU-H71 has undergone several phase Ib clinical trials by Samus Therapeutics [156–158].”

The corresponding references were newly added and the original references 150 and 151 have been replaced by 151–158:

151. Speranza, G.; Anderson, L.; Chen, A.P.; Do, K.; Eugeni, M.; Weil, M.; Rubinstein, L.; Majerova, E.; Collins, J.; Horneffer, Y.; et al. First-in-human study of the epichaperome inhibitor PU-H71: clinical results and metabolic profile. *Investig. New Drugs* **2018**, *36*, 230–239.
152. Rodina, A.; Wang, T.; Yan, P.; Gomes, E.D.; Dunphy, M.P.; Pillarsetty, N.; Koren, J.; Gerecitano, J.F.; Taldone, T.; Zong, H.; et al. The epichaperome is an integrated chaperome network that facilitates tumour survival. *Nature* **2016**, *538*, 397–401.
153. Usmani, S.Z.; Bona, R.D.; Chiosis, G.; Li, Z. The anti-myeloma activity of a novel purine scaffold HSP90 inhibitor PU-H71 is via inhibition of both HSP90A and HSP90B1. *J. Hematol. Oncol.* **2010**, *3*, 40.
154. Patel, H.J.; Patel, H.D.; Ochiana, S.O.; Yan, P.; Sun, W.; Patel, M.R., Shah, S.K.; Tramentozzi, E.; Brooks, J.; Bolaender, A.; et al. Structure–Activity Relationship in a Purine-Scaffold Compound Series with Selectivity for the Endoplasmic Reticulum Hsp90 Paralog Grp94. *J. Med. Chem.* **2015**, *58*, 3922–3943.
155. Huck, J.D.; Que, N.L.S.; Immormino, R.M.; Shrestha, L.; Taldone, T.; Chiosis, G.; Gewirth, D.T.; et al. NECA derivatives exploit the paralog-specific properties of the site 3 side pocket of Grp94, the endoplasmic reticulum Hsp90. *J. Biol. Chem.* **2019**, *294*, 16010–16019.
156. Assess the Safety, Tolerability Oral PU-H71 in Subjects Taking Ruxolitinib. *Identifier* NCT03935555. Available online: https://clinicaltrials.gov/ct2/show/NCT03935555?term=PU-H71&draw=2&rank=1 (accessed on 2 April 2021)
157. PU-H71 with Nab-paclitaxel (Abraxane) in Metastatic Breast Cancer. *Identifier* NCT03166085. Available online: https://clinicaltrials.gov/ct2/show/NCT03166085?term=PU-H71&draw=2&rank=2 (accessed on 2 April 2021)
158. The First-in-human Phase I Trial of PU-H71 in Patients with Advanced Malignancies. *Identifier* NCT01393509. Available online: https://clinicaltrials.gov/ct2/show/NCT01393509?term=PU-H71&draw=2&rank=4 (accesses on 2 April 2021)

The authors mistakenly confused PU-H71 with prior AUY-922 clinical trials and would like to make the following amendments to the published paper:

In the Section 4.1.2, the following statement has been changed to clarify the sentence: “However, studies were discontinued after it failed to show clinically significant effectiveness at the maximum tolerable dose [143].” was changed to “However, these studies were discontinued after it failed to show clinically significant safety or effectiveness [143,144].” 

The authors also note that the corresponding reference 143 was replaced with the following two:

143. Renouf, D.J.; Hedley, D.; Krzyzanowska, M.K.; Schmuck, M.; Wang, L.; Moore, M.J. A phase II study of the HSP90 inhibitor AUY922 in chemotherapy refractory advanced pancreatic cancer. *Cancer Chemother. Pharmacol.* **2016**, *78*, 541–545.
144. Bendell, J.C.; Bauer, T.M.; Lamar, R.; Joseph, M.; Penley, W.; Thompson, D.S.; Spigel, D.R. Owera, R.; Lane, C.M.; Earwood, C.; et al. A Phase 2 Study of the Hsp90 Inhibitor AUY922 as Treatment for Patients with Refractory Gastrointestinal Stromal Tumors. *Cancer Investig.* **2016**, *34*, 265–270.

Meanwhile, the reference citation numbers after [142] in the main text are changed accordingly.

The authors apologize for any inconvenience caused and state that the scientific conclusions are unaffected. The academic editor has checked this correction. The original article has been updated.
